# Impact of intracellular domain flexibility upon properties of activated human 5-HT_3_ receptors*

**DOI:** 10.1111/bph.12536

**Published:** 2014-03-18

**Authors:** J L Kozuska, I M Paulsen, W J Belfield, I L Martin, D J Cole, A Holt, S M J Dunn

**Affiliations:** 1 Department of Pharmacology, University of AlbertaEdmonton, AB, Canada; 2 Theory of Condensed Matter Group, Cavendish Laboratory, University of CambridgeCambridge, UK

**Keywords:** 5-HT_3_ receptor, ligand-gated ion channels, electrophysiology, single-channel conductance, intracellular domain, allostery, cooperativity, constrained geometric simulation

## Abstract

**Background and Purpose:**

It has been proposed that arginine residues lining the intracellular portals of the homomeric 5-HT_3_A receptor cause electrostatic repulsion of cation flow, accounting for a single-channel conductance substantially lower than that of the 5-HT_3_AB heteromer. However, comparison of receptor homology models for wild-type pentamers suggests that salt bridges in the intracellular domain of the homomer may impart structural rigidity, and we hypothesized that this rigidity could account for the low conductance.

**Experimental Approach:**

Mutations were introduced into the portal region of the human 5-HT_3_A homopentamer, such that putative salt bridges were broken by neutralizing anionic partners. Single-channel and whole cell currents were measured in transfected tsA201 cells and in *Xenopus* oocytes respectively. Computational simulations of protein flexibility facilitated comparison of wild-type and mutant receptors.

**Key Results:**

Single-channel conductance was increased substantially, often to wild-type heteromeric receptor values, in most 5-HT_3_A mutants. Conversely, introduction of arginine residues to the portal region of the heteromer, conjecturally creating salt bridges, decreased conductance. Gating kinetics varied significantly between different mutant receptors. EC_50_ values for whole-cell responses to 5-HT remained largely unchanged, but Hill coefficients for responses to 5-HT were usually significantly smaller in mutants. Computational simulations suggested increased flexibility throughout the protein structure as a consequence of mutations in the intracellular domain.

**Conclusions and Implications:**

These data support a role for intracellular salt bridges in maintaining the quaternary structure of the 5-HT_3_ receptor and suggest a role for the intracellular domain in allosteric modulation of cooperativity and agonist efficacy.

**Linked Article:**

This article is commented on by Vardy and Kenakin, pp. 1614–1616 of volume 171 issue 7. To view this commentary visit http://dx.doi.org/10.1111/bph.12550.

## Introduction

**Figure 1:**
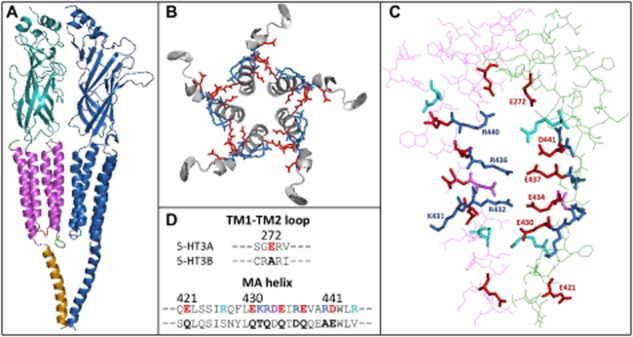
Model of the 5-HT_3_A receptor obtained using the cryo-EM structure of the nACh receptor (PDB 2bg9; Unwin, 2005) as a template. This model was constructed using the conformation of the δ subunit as it exists at the interface with the α subunit and rotated around the channel axis to generate a homomeric channel with fivefold symmetry. (A) Two subunits of the receptor are shown as viewed from a position parallel to the membrane. The subunit on the right is shown in blue, the other subunit is coloured to illustrate distinct regions of the protein; ECD (cyan), TMD (violet) and ICD. The ICD consists of the TM1-TM2 loop (red), the beginning of the TM3-TM4 domain (green) and the MA helix (orange). (B) The MA helix as viewed looking down the ion pore from above the membrane. Other domains were deleted for clarity. Basic amino acid residues (blue) and acidic amino acid residues (red) project into the portal region between subunits. (C) The MA helix of two adjacent subunits as viewed from a position parallel to the membrane. The arginine residues previously identified as important determinants of single-channel conductance (Kelley *et al*., 2003) are highlighted in blue as well as K431, and the glutamate and aspartate residues that were investigated in this study are in red. Other charged residues not investigated are highlighted in cyan (arginine) and violet (aspartate) to illustrate the extent of charged residue presence in this region. A space-filling representation of these portals is shown in Supporting Information Figure S1. (D) A partial sequence alignment of the TM1-TM2 loop and MA helices of the 3A and 3B subunits.

The 5-HT type 3 (5-HT_3_) receptor is a member of the Cys-loop family of pentameric ligand-gated ion channels (pLGICs); this family also includes the GABA type A (GABA_A_) receptor, the glycine receptor and the archetypal family member, the nicotinic ACh (nACh) receptor. Like the nACh receptor, the 5-HT_3_ receptor is a cation-selective channel; though structural data are not available for the 5-HT_3_ receptor, the structure of the nACh receptor from *Torpedo marmorata* has been resolved to 4 Å and comprises five subunits, each with three domains, arranged pseudosymmetrically around the integral ion channel pore (Unwin, 2005). Agonist recognition occurs at the extracellular domain (ECD), while the second (pore-lining) helix of the tetrahelical transmembrane domain (TMD) has been associated with gating. The intracellular domain (ICD) is formed by a short loop separating TM1 from TM2 and by the extensive TM3-TM4 domain. Here, cryo-electron microscopy (cryo-EM) images of the nACh receptor reveal a single membrane-associated (MA) helix from each subunit arranged to form an inverted cone, the base resting against the intracellular membrane. Between the MA helices are portals through which ions access the intracellular space (Figure 1A). With the exception of the TM3-TM4 ICD, these general features are also present in crystal structures of the prokaryotic receptors ELIC (Hilf and Dutzler, 2008) and GLIC (Bocquet *et al*., 2009; in which TM3-TM4 is a short linker) and the eukaryotic GluCl receptor (Hibbs and Gouaux, 2011; in which TM3-TM4 was replaced by a short linker to facilitate crystallization).

Five genes have been identified that encode 5-HT3 subunits A-E, although only the A and B subunits have been significantly characterized. The 5-HT3A subunit, obtained by expression cloning (Maricq *et al*., 1991), can be expressed as a homomer; the 5-HT3B subunit forms heteromeric receptors when expressed with the 3A subunit (Davies *et al*., 1999). Electrophysiological measurements of the homomer expressed in *Xenopus* oocytes reveal an EC_50_ for 5-HT that is almost one order of magnitude lower than that of the heteromeric receptor, and a Hill coefficient that is more than twofold higher.

The conductance of the homomer, when estimated by noise analysis, is less than 1 pS (Brown *et al*., 1998), compared with 17 pS for the heteromer (Davies *et al*., 1999). This marked disparity in conductance has been attributed to three arginine residues, found in the 3A subunit MA helices but absent from those of the 3B subunits that were proposed to cause steric interference or electrostatic repulsion of sodium ion passage through the portals (Kelley *et al*., 2003; Deeb *et al*., 2007). Replacement of these arginines with aligned residues from the B subunit yields a triple mutant, referred to as the 5-HT_3_A(QDA) receptor, in which conductance is increased to a value similar to that of the heteromer (Kelley *et al*., 2003).

No structure has been determined for the 5-HT_3_ receptor. Of those related proteins for which structures are available, ELIC, GLIC and GluCl lack a substantial TM3-TM4 domain, and only the 2bg9 structure of the nACh receptor provides some detail of the MA helix (Unwin, 2005). Thus, a homology model of the 5-HT_3_A receptor, complete with MA helix (Figure 1) was constructed with the 2bg9 structure used as a template. In the primary sequence of the MA helix of the 5-HT3A subunit, the arginine residues (*vide supra*) lie in positions 432, 436 and 440 (Figure 1D), and are separated from each other by approximately a single α-helix turn (Figure 1C). In addition, each arginine is followed, either immediately or two residues distant, by a glutamate or aspartate. This charge distribution is similar to that identified in the nACh receptor (Finer-Moore and Stroud, 1984) but is not present in the 5-HT3B subunit (Figure 1D). When the MA helices are viewed from above (Figure 1B) the basic residues on one helix and the acidic residues on the adjacent helix project towards one another. Viewed from the side (Figure 1C), the model suggests that salt bridges may exist between basic and acidic residues on either side of each portal.

We hypothesized that cation flow in the homomeric receptor was restricted not by steric or electrostatic repulsion, but rather by a structural rigidity of the ICD to which interactions of these charged residues contribute. Thus, the consequence of replacement of charged residues in the 5-HT_3_A(QDA) receptor might extend far beyond a localized electrostatic effect (Kelley *et al*., 2003), to a change in the energy landscape of the protein that influences agonist binding and/or subsequent transduction events. Here, we demonstrate that breaking salt bridges putatively involving residues lining the ICD portals, without removal of the localized positive charges, results in substantial increases in single-channel conductance and changes in gating and cooperativity of agonist binding. Constrained geometric simulations support the view that mutations introduced in the portal region are associated with global changes in protein mobility and receptor conformation. These observations raise intriguing questions regarding the effects on responses to agonists of conformational selection resulting from ICD phosphorylation (McKinnon *et al*., 2012) or association of putative intracellular binding partners with the ICD (Everitt *et al*., 2009; Kenakin, 2013).

## Methods

### DNA constructs and expression in tsA201 cells and *Xenopus* oocytes

Human 5-HT3A and B subunit cDNA were subcloned into the pcDNA3.1(+) expression vector (Invitrogen, San Diego, CA, USA). Mutants were constructed as described previously (Paulsen *et al*., 2009) and confirmed by DNA sequencing. Transfection of tsA201 cells with wild-type or mutant subunit cDNA was performed by electroporation (Nucleofector II, Amaxa Biosystems; ESBE Scientific, Markham, ON, Canada) using 5 μg cDNA and approximately 1 × 10^6^ cells. Cells were used for single-channel recordings (excised outside-out patches) 24–56 h following transfection. Stage V–VI *Xenopus laevis* oocytes were isolated and prepared as previously described (Smith *et al*., 2004). Follicle-free oocytes were microinjected with 50 nL of 1 μg·μL^−1^ wild-type or mutant subunit cRNA. 5-HT_3_A(QDA) and 5-HT_3_A(K431T) receptor cRNAs were diluted 1/50 to reduce expression to a level that would not saturate the amplifier. Injected oocytes were incubated in ND96 buffer (96 mM NaCl, 1.8 mM CaCl_2_, 2 mM KCl, 1 mM MgCl_2_ and 5 mM HEPES, pH 7.4 with NaOH) containing 50 μg·mL^−1^ gentamicin (Gibco, Grand Island, NY, USA) in 96-well plates at 14°C for at least 48 h prior to functional analysis.

### Electrophysiology

The outside-out patch configuration was used to measure single-channel currents from transfected tsA201 cells. Cells were perfused with a solution of (in mM): 140 NaCl, 2.8 KCl, 2 MgCl_2_, 1 CaCl_2_, 10 HEPES, pH 7.4 with NaOH. Thick walled (1.7 mm OD, 0.75 mm ID) 22% PbO glass #0010 (World Precision Instruments, Inc., Sarasota, FL., USA) patch electrodes were pulled with a Flaming Brown P-87 micropipette puller (Sutter Instrument Company, Novato, CA). Patch electrodes had a resistance of 10–15 MΩ when filled with (in mM): 130 K-gluconate, 5 NaCl, 2 MgCl_2_, 5 EGTA, 1 CaCl_2_, 10 HEPES, pH 7.4 with KOH. 5-HT was applied via a diffusion pipette positioned approximately 30 μm from a voltage-clamped cell. Single-channel currents were acquired using Strathclyde electrophysiology software (John Dempster, WinEDR 3.1.0). Currents were filtered at 5 kHz by an NPI LPBF-48DG Bessel filter (LPBF-48DG, NPI Electronic, Tamm, Germany), digitized at 100 kHz and converted to a digital signal (Digidata 1322A, Axon Instruments Inc., Foster City, CA, USA).

5-HT-induced currents were measured in *Xenopus* oocytes by standard two-electrode voltage clamp techniques using a GeneClamp 500B amplifier (Axon Instruments Inc.) at a holding potential of −60 mV. Electrodes were filled with 3 M KCl and had a resistance between 0.3 and 1.5 MΩ in Frog Ringer's solution (110 mM NaCl, 2 mM KCl, 1.8 mM CaCl_2_ and 5 mM HEPES, pH 7.4). Unless otherwise stated, data are reported as mean ± SEM.

### Electrophysiology data analysis

Single-channel traces were analysed with Clampfit 10.3 (Molecular Devices, Sunnyvale, CA, USA) using 50% threshold of current amplitude. A Gaussian distribution was fitted to amplitude histograms using the following formula (Clampfit 10.3):


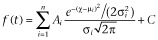


where *n* is the number of components and *A* is the amplitude of the Gaussian mean μ for each component *i*. The Gaussian standard deviation is σ and *C* is the constant *y*-offset. The mean of the amplitude is reported as the main point conductance at a holding potential of −60 mV.

In measuring open and closed durations, events of less than 0.1 ms were ignored since, when filtering at 5 kHz, complete resolution of events shorter than 0.1 ms is not possible. Only traces that could reasonably be believed to contain a single channel were used for the closed duration analysis, that is, at no time during recording was any summation of currents observed. Entire traces were analysed and the resulting open and closed duration histograms were fitted with a maximum-likelihood fit. Corradi *et al*. (2009) reported small differences in the open and closed duration analysis when performed before and after cluster analysis using a critical closed duration to separate these clusters. In light of these reported differences, a cluster analysis was not applied to these data. Due to relatively few closed events longer than 100 ms, the *x*-axis of the closed duration histograms is limited to events shorter than 100 ms. Populations of open and closed durations were fitted using the following formula, accepting the lowest number of best fit parameters:


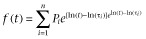


where *P* is the proportion and τ is the time constant for each component *i*.

EC_50_ values were determined from concentration-effect curves obtained in oocytes with 5-HT as agonist. Curves were fitted with non-linear regression (Prism 5.03; GraphPad software, San Diego, CA, USA) to a modification of the Hill equation:


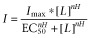


Residual plots were created simultaneously to confirm no systematic deviation of data from the line of best fit, which would be expected if desensitization was compromising measurement of currents at higher agonist concentrations.

### Homology models

Homology models were constructed with the heteromeric nACh receptor structure from *T. marmorata* as template (PDB 2bg9; Unwin, 2005) using Modeller v8.2 (Sali and Blundell, 1993) subsequent to sequence alignment with CLUSTAL W (Thompson *et al*., 1994). The homomeric 5-HT_3_A structure was based on the interface between the α-and δ-subunits. The co-ordinates of R432, R436 and R440 were fitted from the α-subunit onto the δ-subunit, and the resulting δ-subunit was extracted and rotated around the channel axis to produce the homomeric 5-HT_3_A receptor structure with fivefold rotational symmetry. The structure was protonated and minimized in the gas phase using the AMBER11 package (Case *et al*., 2010) before rigidity analysis. Mutant structures were generated in the same manner, except that mutated side chains (E434Q, E437Q and D441N for the QQN mutant, and E434Q) were extracted from their respective homology models for RMS fitting onto the δ-subunit.

### Constrained geometric simulation

Framework rigidity optimized dynamics algorithm (FRODA) is a computationally inexpensive method for sampling protein conformational space that employs a Lagrangian constraints-based approach, outputting an ensemble of structures that obey pre-defined constraints on bond lengths and angles, hydrogen bonds and hydrophobic interactions. FRODA simulations were performed with the floppy inclusions and rigid substructure topography (FIRST)/FRODA version 6.2 software downloaded from http://flexweb.asu.edu/ (Wells *et al*., 2005). FRODA takes, as an input, a decomposition of the protein into rigid and flexible regions provided by the FIRST software. To generate rigid cluster decompositions, the hydrogen bond energy cut-off (Ecut) was set to −1.0 kcal·mol^−1^ and the ‘H3’ hydrophobic tether scheme was employed. The principal component subspaces spanned by FRODA simulations have been shown to be very robust with respect to chosen value of Ecut (David and Jacobs, 2011); we have repeated our simulations with an Ecut value of −0.6 kcal·mol^−1^, observing no difference in qualitative behaviour (Supporting Information Figure S3c).

For FRODA dynamics, a step length of 0.1 Å was employed and the continuous motion method was switched on. For each receptor, 16 independent simulations, each sampling 200 000 conformations, were run. A total of 6400 conformations were stored for analysis. The stereochemical quality of the snapshots was ascertained via PROCHECK (Laskowski *et al*., 1993); for all snapshots, the maximum number of bad contacts was 17 and fewer than 3% of the amino acids were in disallowed regions of the Ramachandran plot. Positional fluctuations about the average structures were calculated by residue using the AMBER11 package and were averaged over all snapshots and the five subunits. Portal width profiles were generated with the HOLE software (Smart *et al*., 1996) by searching for channels along a line joining the centre of the MA helices with the centre of each portal in turn (defined by residues L335, R436, E437 and R444). For each receptor conformation, the five portal widths were determined as the minimum width along the resulting profile. An example HOLE profile through the MA portals is shown in Supporting Information Figure S4.

### Statistical analysis

The amplitude of receptor currents, EC_50_ values and Hill coefficients were compared by one-way anova with Tukey's *post* test. Significance between groups is shown for those different from 5-HT_3_A (QDA), 5-HT_3_A(QQN) and 5-HT_3_AB in the case of the conductance histogram. The open and closed dwell durations were compared by an unpaired *t*-test with Welch's correction.

### Materials

5-HT was purchased from Sigma-Aldrich (St. Louis, MO, USA) and stock solutions (10 mM) were prepared in sterile water. Restriction enzymes and cRNA transcript preparation materials were from Invitrogen (Burlington, ON, Canada), Promega (Madison, WI, USA) or New England Biolabs (Pickering, ON, Canada). *Pfu* Turbo DNA polymerase, for site-directed mutagenesis, was from Stratagene (La Jolla, CA, USA). Custom primers were prepared by IDT (Coralville, IA, USA).

## Results

### Single-channel point conductance of intracellular loop mutants

To test the hypothesis that charged residues lining the portals formed by the MA helices stabilize the quaternary structure of the ICD, we identified three acidic residues located in the portals opposite arginines 432, 436 and 440, and mutated these to their corresponding amides. In doing so, putative salt bridges were disrupted without loss of those arginine residues proposed to be responsible for electrostatic repulsion. The resulting E434Q, E437Q, D441N mutant, referred to as the 5-HT_3_A(QQN) receptor, and the corresponding single-point mutants, were expressed in tsA201 cells, and single-channel conductances were measured. To facilitate comparative studies, the 5-HT_3_A(QDA) receptor, and the heteromeric and homomeric wild-type receptors, were also expressed.

**Figure 2:**
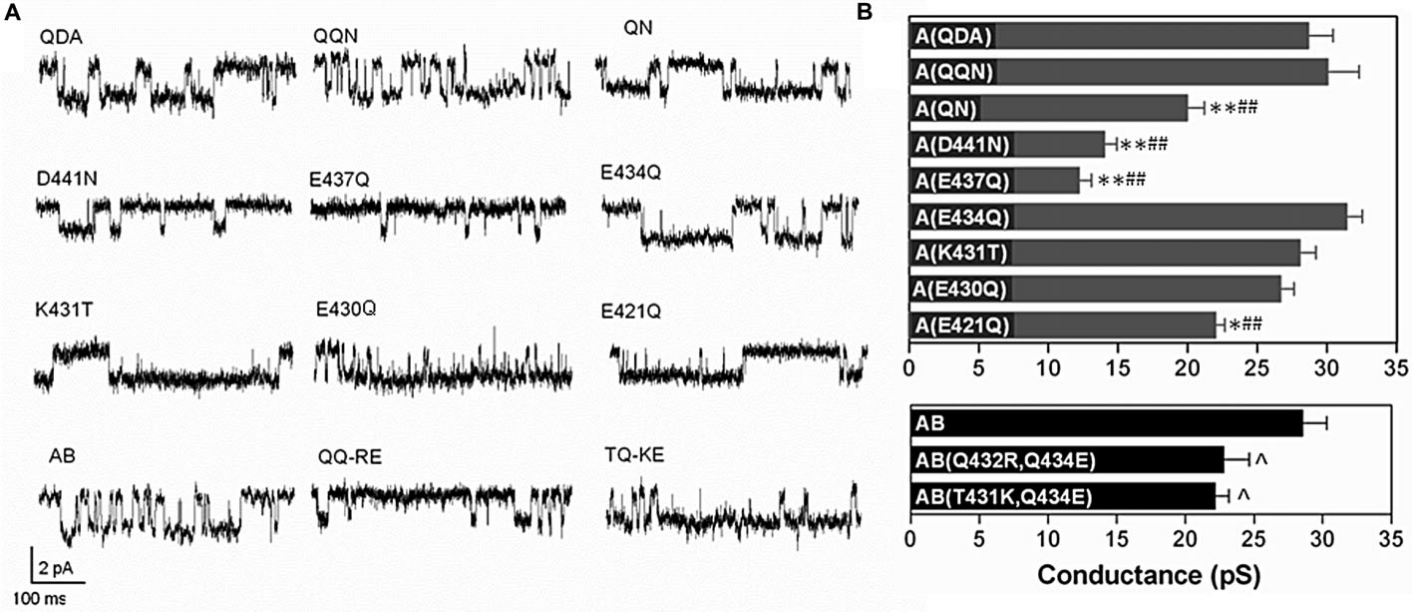
The effect of MA helix mutations on the single-channel conductance of 5-HT_3_A receptors expressed in tsA201 cells. Receptors were expressed in tsA201 cells and outside-out patches were excised from these cells. The holding potential was −60 mV for all receptor constructs and 5-HT (10 μM) was used to evoke channel activity in all the homomeric receptors. 5-HT 100 μM was used to evoke activity of wild-type heteromeric receptors and 5-HT_3_AB(T431K,Q434E) receptors while 1 mM 5-HT was used for 5-HT_3_AB(Q432R,Q434E) receptors, to reflect the difference in 5-HT affinity. (A) Examples of the single-channel currents. Openings are downward deflections. QDA, 5-HT_3_A(QDA) receptor; QQN, 5-HT_3_A(QQN) receptor; QN, 5-HT_3_A(E437Q+D441N) receptor; D441N, 5-HT_3_A(D441N) receptor; E437Q, 5-HT_3_A(E437Q) receptor; E434Q, 5-HT_3_A(E434Q) receptor; K431T, 5-HT_3_A(K431T) receptor; E430Q, 5-HT_3_A(E430Q) receptor; E421Q, 5-HT_3_A(E421Q) receptor; AB, 5-HT_3_AB receptor; QQ-RE, 5-HT_3_AB(Q432R,Q434E) receptor and TQ-KE, 5-HT_3_AB(T431K,Q434E) receptor. (B) A comparison of the point conductance of each 5-HT_3_ receptor. Data are reported as mean ± SEM from at least three independent experiments. An anova was done to determine statistical differences from the 5-HT_3_A(QDA) receptor (*P < 0.05, **P < 0.01), the 5-HT_3_A(QQN) receptor (^#^*P* < 0.05, ^##^*P* < 0.01) and the 5-HT_3_AB receptor (∧*P* < 0.05).

Unlike the wild-type 5-HT_3_A (homomeric) receptor, the single-channel conductance of the 5-HT_3_A(QQN) receptor could be resolved at a holding potential of −60 mV. The mean point conductance was 30.1 ± 2.3 pS and, under identical recording conditions, was not significantly different from that of the 5-HT_3_A(QDA) receptor (28.7 ± 1.8 pS). Neither were significantly different from the heteromeric receptor conductance (28.5 ± 1.8 pS), although all three values differed somewhat from previously-published conductances (Davies *et al*., 1999; Kelley *et al*., 2003; Reeves *et al*., 2005). Examples of single-channel recordings are shown in Figure 2A and the mean point conductance obtained with each mutant at −60 mV is shown in Figure 2B. Examples of extended recordings, along with amplitude histograms, are provided in Supporting Information Figure S2.

The three acidic residues mutated to yield the 5-HT_3_A(QQN) receptor were also mutated individually, or in pairs, and mean point conductances were measured. The 5-HT_3_A(D441N) receptor had a main conductance of 14.0 ± 0.9 pS while that of the 5-HT_3_A(E437Q) receptor was 12.2 ± 0.9 pS. Both are smaller than conductances determined for the 5-HT_3_A(QDA) and 5-HT_3_A(QQN) receptors (*P* < 0.01). The 5-HT_3_A(E437Q, D441N) receptor opens to a main conductance state of 19.9 ± 1.2 pS, illustrating that the double mutation enhanced channel conductance to a greater degree than did the individual corresponding single mutations. This conductance is still less than that determined for the 5-HT_3_A(QQN) receptor (*P* < 0.01). From our model, E272, located in the TM1-TM2 loop, appears to lie close enough to R440 to establish a salt bridge interaction. However, single-channel currents of the 5-HT_3_A(E272Q) receptor could not be resolved.

The 5-HT_3_A(E434Q) receptor opens to a main state of 31.4 ± 1.1 pS, which is not significantly different from those determined for the 5-HT_3_A(QQN) or 5-HT3A(QDA) receptors. In light of this unexpected observation, three further charged residues upstream of E434 were mutated in an attempt to identify other potential salt bridge partners at the apex of the MA helices. The K431T mutation resulted in a receptor that opens with a conductance of 28.1 ± 1.2 pS and the 5-HT_3_A(E430Q) receptor has a conductance of 26.7 ± 1.0 pS; neither are significantly smaller than those of the 5-HT_3_A(QQN) nor 5-HT_3_A(QDA) receptors. Receptors bearing the E421Q mutation open with a conductance of 22.0 ± 0.7 pS. These data suggest that charge interactions involving E421, E430, K431 and E434 play a major role in limiting conductance by tethering the five MA helices to create a rigid apex.

The converse experiment was performed, to investigate whether introducing a pair of charged residues in the apex of the MA helix in the heteromeric receptor decreases the conductance of this receptor. 5-HT_3_AB(Q432R, Q434E) and 5-HT_3_AB(T431K, Q434E) receptors were expressed and the mean point conductances were 22.8 ± 1.8 pS and 22.2 ± 1.0 pS respectively. These values are approximately 20% smaller than the conductance of the heteromeric wild-type receptor (*P* < 0.05).

### Consequences of MA helix mutations on open and closed dwell durations

Both the 5-HT_3_A(QQN) and 5-HT_3_A(QDA) receptors display four populations of open durations and three populations of closed durations when activated with 10 μM 5-HT (Table 1, Supporting Information Figure S5). Each open state of the 5-HT_3_A(QQN) receptor has a longer dwell time than the corresponding open state of the 5-HT_3_A(QDA) receptor. While the lengths of the brief open durations were not significantly different, the short, intermediate and long open durations of the 5-HT_3_A(QQN) receptor were significantly longer than the respective 5-HT_3_A(QDA) receptor openings, suggesting that gating of these two receptors is distinct. Of the closed durations of the 5-HT_3_A(QQN) receptor, the short and intermediate closures were significantly shorter than those of the 5-HT_3_A(QDA) receptor.

**Table 1:** Open and closed dwell durations of 5-HT_3_ receptors expressed in tsA201 cells

Receptor	*O*_b_ (ms)	*O*_s_ (ms)	*O*_i_ (ms)	*O*_l_ (ms)	*C*_b_ (ms)	*C*_s_ (ms)	*C*_i_ (ms)
	Area (%)						
5-HT_3_A(QDA)	0.20 ± 0.08	3.01 ± 0.23##	17.98 ± 0.22###	80.44 ± 0.47###	0.21 ± 0.05	3.31 ± 0.15	29.42 ± 0.21###
(*n* = 6)	41.07 ± 2.00	18.59 ± 3.24#	30.80 ± 4.17	9.54 ± 4.83	63.59 ± 1.92###	21.27 ± 2.08	15.15 ± 2.01
5-HT_3_A(QQN)	0.40 ± 0.13	5.00 ± 0.30**	27.07 ± 0.40***##	140.98 ± 0.29***###	0.38 ± 0.09	2.52 ± 0.17*##	23.93 ± 0.26***###
(*n* = 3)	32.67 ± 2.77	22.75 ± 5.38	24.07 ± 5.56	20.51 ± 5.69	53.03 ± 3.41#	31.40 ± 3.40	15.57 ± 2.67
5-HT_3_AB	0.31 ± 0.10	4.53 ± 0.17	31.55 ± 0.20	256.10 ± 1.12	0.28 ± 0.11	4.02 ± 0.24	34.91 ± 0.33
(*n* = 4)	35.19 ± 2.37	30.33 ± 3.47	30.25 ± 3.59	4.22 ± 3.10	37.45 ± 2.81	23.30 ± 3.64	25.15 ± 5.60
5-HT_3_AB(Q432R,Q434E)	0.25 ± 0.06	2.34 ± 0.10###	17.78 ± 0.10###	89.13 ± 0.20###	0.18 ± 0.05	2.55 ± 0.10##	28.95 ± 0.14###
(*n* = 4)	31.33 ± 1.29	24.09 ± 1.55	32.25 ± 2.13	12.33 ± 2.35	47.45 ± 1.60#	22.67 ± 1.52	22.62 ± 2.15
5-HT_3_AB(T431K,Q434E)	0.25 ± 0.05	2.97 ± 0.07##•	21.37 ± 0.17###•••		0.22 ± 0.07	2.85 ± 0.13#	23.84 ± 0.14###•••
(*n* = 4)	44.69 ± 1.48#•	41.01 ± 1.79#••	14.30 ± 1.81#••		48.75 ± 2.09#	28.89 ± 2.32	22.36 ± 2.28

Open and closed duration histograms were constructed from entire single-channel recordings from at least three patches. Histograms were fitted by four components for the open durations [*O*_b_ (brief), *O*_s_ (short), *O*_i_ (intermediate) and *O*_l_ (long)] and three components for the closed durations [*C*_b_ (brief), *C*_s_ (short) and *C*_i_ (intermediate)]. Results are shown as the mean duration ± SEM. A series of unpaired *t*-tests with Welch's correction was used to compare the *τ* and area values of the 5-HT_3_A(QDA) receptor to those of the 5-HT_3_(QQN) receptor

* (*P* < 0.05,

** *P* < 0.01,

*** *P* < 0.001).

In addition, this analysis was used to compare these same parameters of the 5-HT_3_AB receptor with the 5-HT_3_A(QDA) receptor, the 5-HT_3_A(QQN) receptor, the 5-HT_3_AB(Q432R,Q434E) receptor and the 5-HT_3_AB(T431K,Q434E) receptor

# (*P* < 0.05,

## *P* < 0.01,

### *P* < 0.001)

and the 5-HT_3_AB(Q432R,Q434E) receptor to the 5-HT_3_AB(T431K,Q434E) receptor

• (*P* < 0.05,

•• *P* < 0.01,

••• *P* < 0.001).

The open durations of the heteromeric receptor were approximately 0.3, 4.5, 30 and 250 ms when activated with 100 μM 5-HT. Adding a pair of charged residues to the B subunit [Q432R and Q434E (1 mM 5-HT) or T431K and Q434E (100 μM 5-HT)] resulted in open and closed dwell durations shorter than those of the wild-type heteromeric receptor. Thus, removal of charged residues from the homomeric receptor and introduction of charged residues into the heteromeric receptor both affected gating of the channel.

### Concentration response to 5-HT in MA helix mutants

Concentration-response curves to 5-HT were determined in *Xenopus* oocytes for all mutants (Supporting Information Figure S6). The effects of mutations of the wild-type 5-HT_3_A receptor on EC_50_ values for activation by 5-HT were generally small, and with the exception of that for activation of the 5-HT_3_A(QQN) receptor, were not statistically significant. However, introduction of basic residues into the portal region of the 5-HT_3_AB heteromer resulted in significant increases in EC_50_ values (Table 2).

**Table 2:** Relative affinities of 5-HT for 5-HT_3_ receptors expressed in *Xenopus* oocytes

Receptor	Log EC_50_ ± SEM, M	*n*	EC_50_ (95% CI) μM	*n*_H_ ± SEM
5-HT_3_A	−5.75 ± 0.06##	7	1.77 (1.32–2.34)	2.67 ± 0.27##
5-HT_3_A(QDA)	−5.63 ± 0.06##	4	2.35 (1.82–3.02)	2.24 ± 0.23##
5-HT_3_A(QQN)	−5.48 ± 0.04*##	5	3.32 (2.75–3.98)	1.50 ± 0.09**
5-HT_3_A(E437Q,D441N)	−5.56 ± 0.04##	3	2.74 (2.29–3.24)	1.85 ± 0.18*#
5-HT_3_A(D441N)	−5.50 ± 0.08##	4	3.14 (2.14–4.57)	1.57 ± 0.18**
5-HT_3_A(E437Q)	Currents too small			
5-HT_3_A(E434Q)	−5.59 ± 0.07##	4	2.59 (1.91–3.55)	1.50 ± 0.12**
5-HT_3_A(K431T)	−6.03 ± 0.02##	5	0.93 (0.85–1.02)	3.45 ± 0.20*##
5-HT_3_A(E430Q)	−5.65 ± 0.04##	5	2.24 (1.86–2.69)	2.38 ± 0.13##
5-HT_3_A(E421Q)	−5.52 ± 0.04##	5	2.81 (2.57–3.63)	1.57 ± 0.17**
5-HT_3_A(E272Q)	−5.60 ± 0.01##	4	2.50 (2.34–2.63)	2.31 ± 0.20##
5-HT_3_AB	−5.06 ± 0.07**	6	8.65 (6.17–12.02)	1.10 ± 0.11**
5-HT_3_AB(Q432R,Q434E)	−4.21 ± 0.12**##	5	61.55 (35.48–104.71)	0.69 ± 0.03##
5-HT_3_AB(T431K,Q434E)	−4.55 ± 0.07**##	4	28.30 (20.89–38.02)	0.73 ± 0.08#

Data are expressed as mean ± SEM from at least three independent experiments. An anova was done to compare each mutant receptor with the wild-type 5-HT_3_A receptor and the wild-type 5-HT_3_AB receptor

* (*P* < 0.05,

** *P* < 0.01 compared with the 5-HT_3_A receptor;

# *P* < 0.05,

## *P* < 0.01 compared with the 5-HT_3_AB receptor).

In contrast, most mutations of the wild-type 5-HT_3_A receptor resulted in substantial and significant decreases in the Hill coefficient for concentration-dependence of 5-HT-mediated receptor activation (Table 2), although the Hill coefficient in the 5-HT_3_A(K431T) mutant was increased, compared with that in the wild-type receptor. In the heteromeric receptor, introduction of basic residues resulted in modest but significant decreases in Hill coefficients, to values less than unity. These data may be consistent with an allosteric effect of mutations introduced within the MA helices on cooperativity of agonist binding at sites located in the ECD, around 100 Å from the intracellular portal region, or on the ability of the protein to change conformation in response to agonist binding with no requirement for a change in agonist *K*_D_ (Colquhoun, 1998).

Due to the slow perfusion rate and the slow response of the clamp, desensitization can limit the peak measured current in oocytes, particularly at high agonist concentrations, thereby affecting EC_50_ and Hill coefficient determinations. Residual plots of concentration-response curves (Supporting Information Figure S6) confirmed that data showed no pattern of variation from the line of best fit that would indicate desensitization.

### Flexibility in 5-HT_3_A, 5-HT_3_A(QQN) and 5-HT_3_A(E434Q) receptors

Disruption of salt bridges in the MA helices presumably modifies the network of constraints in the 5-HT_3_A receptor and, hence, increases the number of degrees of freedom available during constrained motion. Decomposition of the modelled wild-type 5-HT_3_A receptor into rigid and flexible regions is shown in Supporting Information Figure S3a and reveals a flexible ECD, while the TMD and MA helices form large rigid clusters. E434 and E437 form rigid inter-helix clusters with R432 and R436 via salt bridges, while D441 and R444 form a similar intra-helix cluster (Supporting Information Figure S3b).

**Figure 3:**
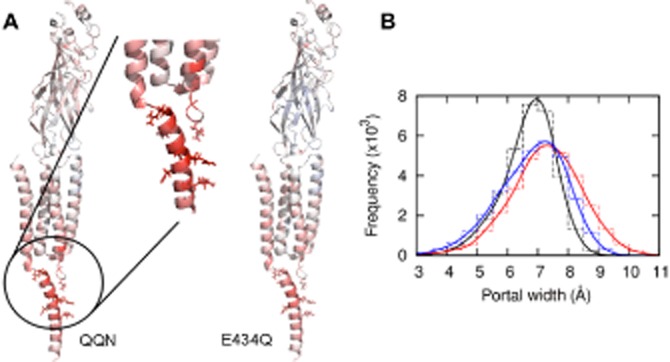
Flexibility in 5-HT_3_A, 5-HT_3_A(QQN) and 5-HT_3_A(E434Q) receptors. (A) One subunit of each 5-HT_3_A mutant receptor is coloured by root mean square fluctuation relative to wild type (red denotes increased and blue decreased flexibility). Large increases in flexibility are observed at the top of the MA helices, close to the mutation sites and at the base of the TM3 helix (up to 0.4 Å) (inset). Figures generated using Pymol (The PyMOL Molecular Graphics System, Version 1.3 Schrödinger, LLC). (B) Distribution of portal widths for the three receptors from constrained geometric simulation. Histograms are fitted with cubic splines. The QQN (red lines) and E434Q (blue lines) mutant receptor portals are, on average, wider and fluctuate in diameter more than the wild-type 5-HT_3_A receptor portals (black lines).

To determine whether or not the loss of constraints caused by the removal of all three salt bridge clusters in the QQN mutant increases MA helix mobility, the rigidity analysis was used as input to FRODA constrained geometric simulation (Wells *et al*., 2005). Indeed, after sampling an ensemble of receptor conformations that maintain rational bonding and steric constraints, residues of the MA helices in the QQN mutant show larger root mean square fluctuations than those of the 5-HT_3_A receptor (Figure 3A; Supporting Information Figure S7; Supporting Information Videos S1–S3). Though the higher mobility is mostly concentrated at the top of the MA helices, at the sites of the mutations, fluctuations are also transmitted to the rest of the protein via the base of the TM3 helices. Similar behaviour is also observed in simulations of the E434Q mutant. In both cases, this increased mobility also allows for a more extended portal in the mutant receptors compared with the wild type (Figure 3B).

It is important to note that these flexibility predictions provide no information regarding the degree of structural movement in response to agonist binding.

## Discussion

The TM3-TM4 loop of the ICD is the least-conserved region within the pLGIC family and is important in receptor assembly, targeting, trafficking and clustering of family members (for reviews, see Connolly, 2008; Millar and Harkness, 2008). However, the MA helix is the only structural element resolved in the cryo-EM studies of the archetypal member of the family, the nACh receptor (Unwin, 2005). The portals, framed by adjacent MA helices, provide access from the ion channel to the intracellular space, and appear to have a particular importance in restricting channel conductance of the homomeric 5-HT_3_A receptor (Kelley *et al*., 2003), a property that appears of limited relevance in other receptors within the family (Peters *et al*., 2010). Recent studies have also characterized the importance of these portals for the inward rectification exhibited by the homomeric 5-HT_3_A receptor (Baptista-Hon *et al*., 2013). Using a series of truncation mutants, these authors have shown that the unstructured segment of this loop requires >70 amino acids in order to stabilize the structure of the portals delineated by the MA helices: further deletions relieve the sub-pS conductance and the inward rectification of this receptor (Baptista-Hon *et al*., 2013).

In the studies reported here, a homology model of the 5-HT_3_A receptor MA helix was constructed based upon the nACh receptor (Unwin, 2005; Figure 1). With the caveat that the influence of the unresolved region of the TM3-TM4 loop on MA helix structure cannot be accounted for, this model suggests that the quaternary structure of the MA helices is stabilized by inter-and intra-helical salt bridges. The pattern of charged residues suggests that putative salt bridges should be broken by both the QDA and QQN mutations; we demonstrate that the 5-HT_3_A(QDA) and 5-HT_3_A(QQN) receptors have similar single-channel conductances of approximately 30 pS, much higher than that of the wild-type receptor.

The conductance of the single-point E434Q mutant was not different from that of the 5-HT_3_A(QQN) receptor, suggesting that E434 may play a central role in stabilizing the 5-HT_3_A receptor MA helix. FRODA simulations predicted that neutralizing E434 would increase mobility of the MA helices and widen the portals between them, although it is assumed that the mutations introduced do not result in widespread disruption of inter-helical bonding that may further widen the portals.

Since E434 lies closer to the apex of the helices than to the membrane, and close to several other potential salt bridge partners, effects of removing other proximate charges were investigated. The K431T, E430Q and E421Q mutations all increased conductance significantly. However, E430, K431 and R432 represent almost an entire turn of the helix, raising the possibility that one or more of these residues project towards, and interact with, undefined residues in the unresolved section of the TM3-TM4 loop. Such interactions may further stabilize the apex of the cone formed by the five MA helices. Beyond this, informed speculation is precluded by the lack of additional structural information within the ICD of the 5-HT_3_A receptor, which is thought to be disordered (Kukhtina *et al*., 2006).

Mutation of charged residues close to the membrane also increased channel conductance, but to a lesser extent compared with mutation of residues closer to the apex. The model suggests that E272, in the TM1-TM2 linker, is sufficiently close to R440 to support a salt bridge interaction. However, neutralizing E272 (E272Q) did not allow for resolution of single-channel currents, either because the mutation did not increase channel conductance or because the receptor failed to express in cells. Nevertheless, it did express in *Xenopus* oocytes, and the EC_50_ and Hill coefficient for responses to 5-HT were not significantly different from those measured in the wild-type homomer (Table 2). Of course, the model may be compromised in this region and no salt bridge may exist. Indeed, recent work by Hibbs and Gouaux (2011) indicates that in several regions, including the E272-containing TM1-TM2 linker region, structural dissimilarities are apparent when the truncated GluCl channel is compared with the nACh receptor 2bg9 structure. Whether or not these differences would be observed if 2bg9 could be compared with the intact GluCl receptor is open to question. A role for the ICD in allosteric regulation of receptor function, confirmed by our data, implicates displacement of residues beyond the ICD as a consequence of events at the level of the MA helix; truncation of the TM3-TM4 region in GluCl may thus affect structure in other regions of the protein.

Kinetic analysis of the wild-type 5-HT_3_A receptor is not possible due to its lack of a unitary conductance; we therefore compared kinetics of 5-HT_3_A(QQN) with those of the 5-HT_3_A(QDA) receptor. A detailed kinetic analysis of 5-HT_3_A(QDA) has been conducted previously (Corradi *et al*., 2009), albeit under different recording conditions, but our data are very similar. Although the conductances of the 5-HT_3_A(QDA) and 5-HT_3_A(QQN) receptors are comparable, it is clear that the two sets of mutations produce distinct receptors. For each open state, the duration of channel opening is longer for the 5-HT_3_A(QQN) receptor than for the 5-HT_3_A(QDA) receptor, but shorter than for the wild-type heteromer. The 5-HT_3_A(QQN) receptor also has shorter closed durations than does the 5-HT_3_A(QDA) receptor. These observations suggest that the gating efficacy of the 5-HT_3_A(QQN) receptor is greater than that of the 5-HT_3_A(QDA) receptor. Introducing two oppositely charged residues in the MA helix apex of the heteromer B subunit resulted in receptors with decreased conductance and shorter open durations compared with wild-type heteromer, suggesting a decrease in gating efficacy.

With the 5-HT_3_ receptor, differences in Hill coefficients between the homomeric (2.67) and heteromeric (1.1) receptor reflect the higher number of agonist molecules thought to be required to evoke maximal activation of the former (Moura Barbosa *et al*., 2010). Although conductance of the 5-HT_3_A(QDA) receptor is distinct from that of the wild-type parent, Hill coefficients are indistinguishable. This is not the case for the 5-HT_3_A(QQN) receptor, which differs in both conductance and Hill coefficient from the wild type. Parallels for these observations can be found in studies in which the TM3-TM4 loop was replaced by the heptapeptide sequence from GLIC, which affected neither potency nor Hill coefficient observed with 5-HT (Jansen *et al*., 2008). However, incorporation of enhanced cyan fluorescent protein at the ICD significantly reduced the Hill coefficient (Ilegems *et al*., 2004) and also provided evidence of agonist-stimulated fluorescence resonance energy transfer between subunits forming the ICD (Ilegems *et al*., 2005). These data, and data presented herein, support bidirectional communication between the ICD and TMD/ECD of this allosteric protein.

Agonist trafficking in GPCRs demands bidirectional allosteric communication between the extracellular agonist binding sites and intracellular sites with which signal transduction partners associate (Kenakin, 2012). The changes we observed in agonist cooperativity in the 5-HT_3_A(QQN) mutant, compared with the wild-type homomer, highlight the potential for modulation of responses to agonist binding mediated through associations of the ICD with cytosolic binding partners. Indeed, altered mobility of the cytoplasmic ends of the TM3 and TM4 transmembrane segments have been suggested to account for effects upon channel function of phosphorylation or association of proteins with the ICD (McKinnon *et al*., 2012). In this regard, the facilitation of benzodiazepine-mediated potentiation of GABAergic signalling observed in the presence of an intracellular protein, GABA_A_ receptor-associated protein (Everitt *et al*., 2009), may offer a remarkable snapshot of ‘biased agonism’ in a pLGIC family member.

The work presented here has shown that mutations of acidic residues within the MA helices of the wild-type 5-HT_3_A receptor result in similar increases in unitary conductance as were demonstrated previously to occur as a result of arginine neutralizations (Kelley *et al*., 2003). However, the QQN and QDA mutant receptors are distinct from one another, despite similar unitary conductances. We show they are differentiated by their kinetic fingerprints and their disparate cooperativities to agonist activation, and suggest that the MA helices are allosterically linked with the agonist recognition domains and/or with the receptor gating domain, thought to lie in the TMD close to the portal region. We hypothesized that inter-and intra-subunit salt bridges within the five MA helices provide structural rigidity to the inverted cone formed by the helices. This was supported by constrained geometric simulations that suggested a modest increase in mobility when these salt bridges were removed, albeit in a model where our understanding of structure in the TM3-TM4 domain is unavoidably compromised. We propose that this rigidity is responsible for the limited conductance of the wild-type 5-HT_3_A receptor, and that the enhanced flexibility of the mutants alters the energy landscape of the protein and allows for conformations that respond to agonist binding with modified gating, cooperativity and conductance. The introduction of equivalent constructs within the heteromeric receptor imparts changes in conductance, agonist potency and cooperativity, further supporting an involvement of the ICD in allosteric communication between the portal region and critical residues in the TMD and/or ECD of the 5-HT_3_A receptor. The nature and location of residues that may relay signals indicating displacement of the ICD to the gating region of the receptor will be the subject of a future communication.

## Abbreviations

cryo-EM: cryo-electron microscopy
ECD: extracellular domain
Ecut: hydrogen bond energy cut-off
FIRST: floppy inclusions and rigid substructure topography
FRODA: framework rigidity optimized dynamics algorithm
ICD: intracellular domain
MA helix: membrane-associated helix
nACh: nicotinic ACh (receptor)
pLGIC: pentameric ligand-gated ion channel
TM: transmembrane
TMD: transmembrane domain

## Supporting information

http://dx.doi.org/10.1111/bph.12536

Figure S1 Surface renderings of the 5-HT_3_A homology modelled portals. The solvent accessible surface was constructed using WebLab ViewerLite 4.0. The surface is rendered transparent in order that the orientation of the molecule can be discerned within the surface; the side chains are colour coded as in Figure 1C. Images of the portal are viewed from both inside the inverted cone formed by the MA helices (a), and from outside (b), in each case perpendicular to the long axis of the molecule.

Figure S2 The effect of MA helix mutations on the single channel conductance of 5-HT_3_A receptors expressed in tsA201 cells. Outside-out patches were excised from cells. The holding potential was –60 mV for all receptor constructs; 5-HT (10 μM) was used to evoke channel activity. Examples of the single channel currents (a) are shown with openings as downward deflections. The amplitude histograms from a single patch (b) are shown with amplitude (pA) plotted on the *x*-axis and number of events plotted on the *y*-axis. Conductance was calculated from the average current amplitude from at least three patches and from a minimum of two separate transfections.

Figure S3 Rigidity analysis of 5-HT_3_A receptor. (a) Tertiary structure of the 5-HT_3_A receptor with residues coloured by rigid clusters assigned by the FIRST software. The ECD is relatively flexible, while the TMD and MA helices form large rigid units. (b) Close-up of the portal region. E434, E437 and D441, along with their salt bridge partners, are shown as sticks and form small side chain rigid units, spanning the MA helices in the first two cases. L335 is also shown as the fourth residue lining the portal. The dashed circle denotes the approximate position of the portal. (c) Distribution of portal widths for the three receptors from constrained geometric simulation with Ecut set to-0.6 kcal mol^−1^. Histograms are fitted with cubic splines. Decreased flexibility leads to narrower portals (compared to Ecut =-1.0 kcal mol^−1^), while the QQN (red lines) and E434Q (blue lines) mutant receptor portals remain wider than the wild type 5-HT_3_A receptor portals (black lines).

Figure S4 HOLE profiles of the MA portals. (a) Example HOLE channel used to calculate the portal width. Channels through the MA helices are searched for along a line joining the centre of the helices with the centre of a portal (defined by residues L335, R436, E437 and R444, shown as spheres). (b) The channel width is measured at each point along the HOLE profile and the minimum width (denoted by arrows) is assigned to the portal width. The process is repeated for every saved conformation.

Figure S5 Open and closed dwell duration histograms of the (A) 5-HT_3_A(QDA) receptor, (B) 5-HT_3_A(QQN) receptor, (C) 5-HT_3_AB receptor, (D) 5-HT_3_AB(Q432R,Q434E) receptor, (E) 5-HT_3_AB(T431K,Q434E) receptor. 5-HT 10 μM was used to evoke 5-HT_3_A(QDA) receptor and 5-HT_3_A(QQN) receptor channel activity. 5-HT 100 μM was used to evoke activity of wild type heteromeric receptors and 5-HT_3_AB (T431K,Q434E) receptors while 1 mM 5-HT was used for 5-HT_3_AB(Q432R,Q434E) receptors. Open dwell histograms are on the left and closed dwell histograms are on the right.

Figure S6 Concentration-response curves of 5-HT activated 5-HT_3_ receptors expressed in *Xenopus* oocytes. Inserts show the resulting residual plots obtained from the corresponding concentration-response curve. The data variability confirms there is no pattern consistent with desensitization. Receptor populations: (a) 5-HT_3_A wild type; (b) 5-HT_3_A(QDA); (c) 5-HT_3_A(QQN); (d) 5-HT_3_A(E437Q, D441N); (e) 5-HT_3_A(D441N); (f) 5-HT_3_A (E434Q); (g) 5-HT_3_A(K431T); (h) 5-HT_3_A(E430Q); (i) 5-HT_3_A(E272Q); (j) 5-HT_3_AB wild type; (k) 5-HT_3_AB(Q432R,Q434E) and (l) 5-HT_3_AB(T431K,Q434E).

Figure S7 Mutant mobility by residue. Root mean square (RMS) fluctuations of the QQN mutant receptor (red) and E434Q mutant receptor (blue) relative to wild type 5-HT_3_A receptor by residue. Large increases in flexibility are observed at the top of the MA helices, close to the mutation sites, and at the base of the TM3 helix.

Video S1 Examples of conformations generated by FRODA constrained geometric simulations of the MA portal regions of the wild type 5-HT_3_A receptor. Colours denote rigid units generated by the FIRST software. Movies generated using Pymol (The PyMOL Molecular Graphics System, Version 1.3 Schrödinger, LLC).

Video S2 Examples of conformations generated by FRODA constrained geometric simulations of the MA portal regions of the 5-HT_3_A(QQN) receptor. Colours denote rigid units generated by the FIRST software. Movies generated using Pymol (The PyMOL Molecular Graphics System, Version 1.3 Schrödinger, LLC).

Video S3 Examples of conformations generated by FRODA constrained geometric simulations of the MA portal regions of the 5-HT_3_A(E434Q) receptor. Colours denote rigid units generated by the FIRST software. Movies generated using Pymol (The PyMOL Molecular Graphics System, Version 1.3 Schrödinger, LLC).
