# Supplementary figures and images for: Impact of intracellular domain flexibility upon properties of activated human 5-HT3 receptors
Source: Br J Pharmacol. 2014 Mar 18;171(7):1617–28. doi: 10.1111/bph.12536 (PMC3966743; doi:10.1111/bph.12536)

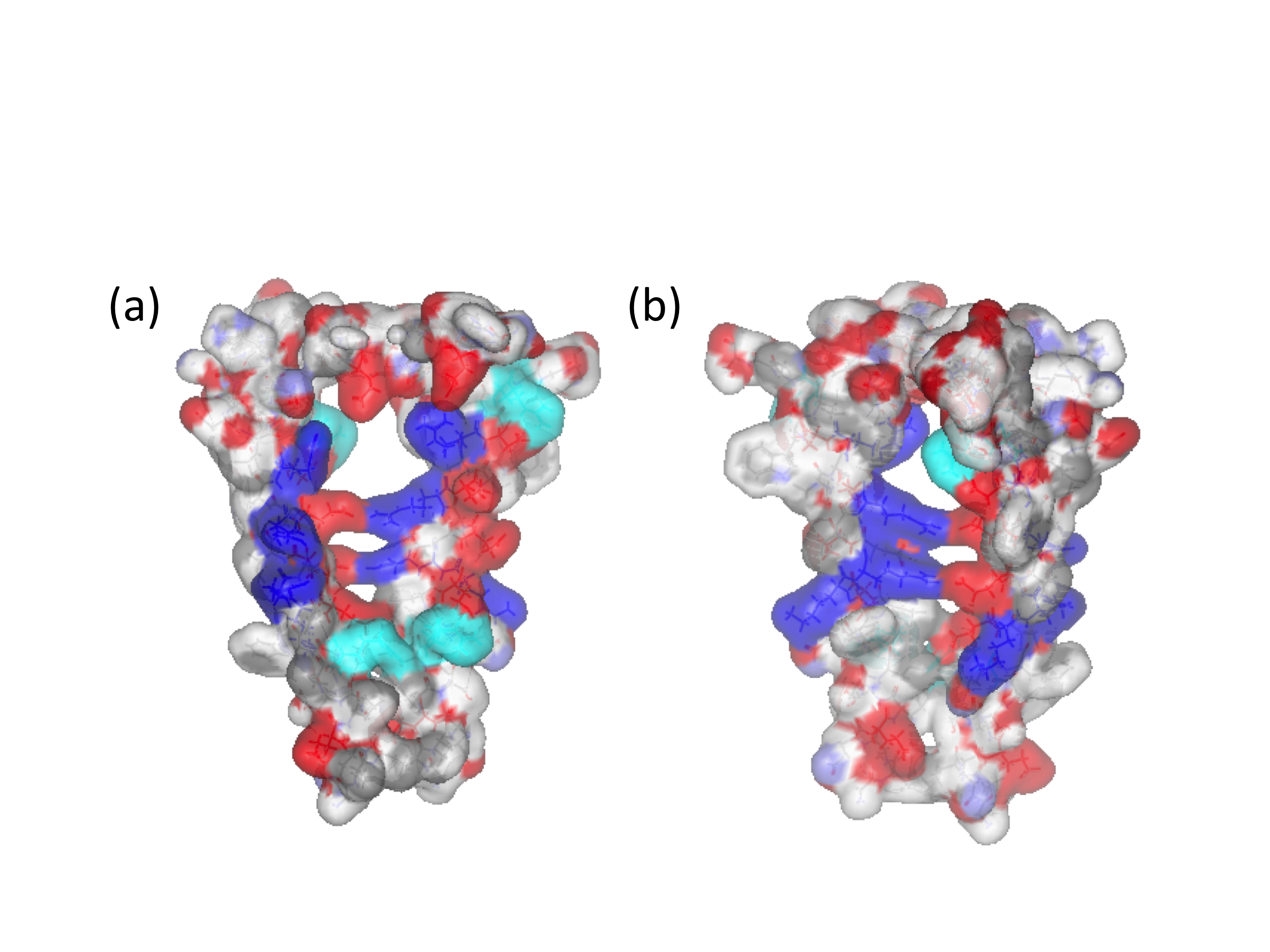

Supplement: Figure S1 — Surface renderings of the 5-HT3A homology modelled portals. The solvent accessible surface was constructed using WebLab ViewerLite 4.0. The surface is rendered transparent in order that the orientation of the molecule can be discerned within the surface; the side chains are colour coded as in Figure 1C. Images of the portal are viewed from both inside the inverted cone formed by the MA helices (a), and from outside (b), in each case perpendicular to the long axis of the molecule. [file bph0171-1617-sd1.tif]

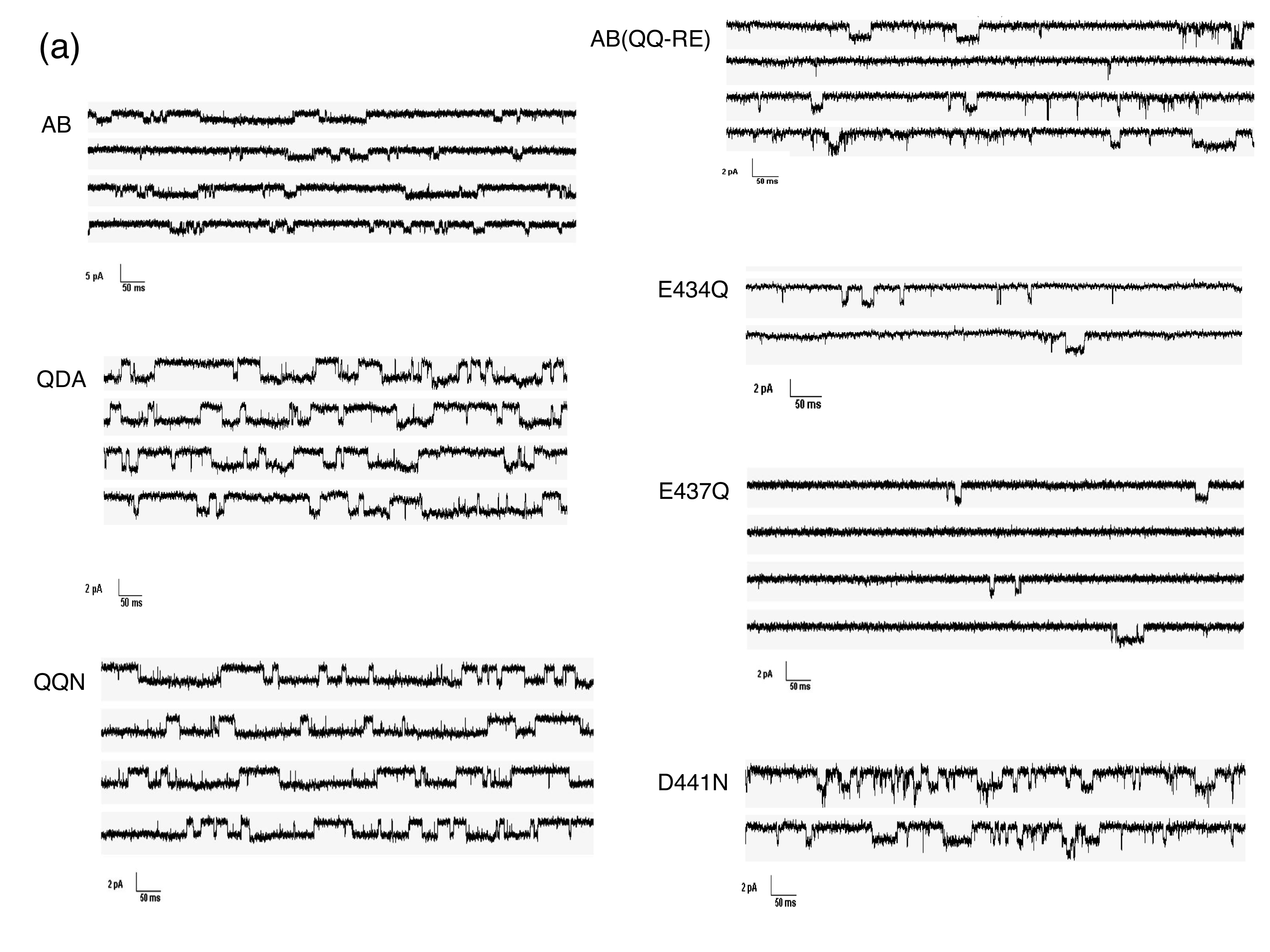

Supplement: Figure S2 — The effect of MA helix mutations on the single channel conductance of 5-HT3A receptors expressed in tsA201 cells. Outside-out patches were excised from cells. The holding potential was –60 mV for all receptor constructs; 5-HT (10 μM) was used to evoke channel activity. Examples of the single channel currents (a) are shown with openings as downward deflections. The amplitude histograms from a single patch (b) are shown with amplitude (pA) plotted on the x-axis and number of events plotted on the y-axis. Conductance was calculated from the average current amplitude from at least three patches and from a minimum of two separate transfections. [file bph0171-1617-sd2.zip › bph_12536_sm_Figure S2a.tiff]

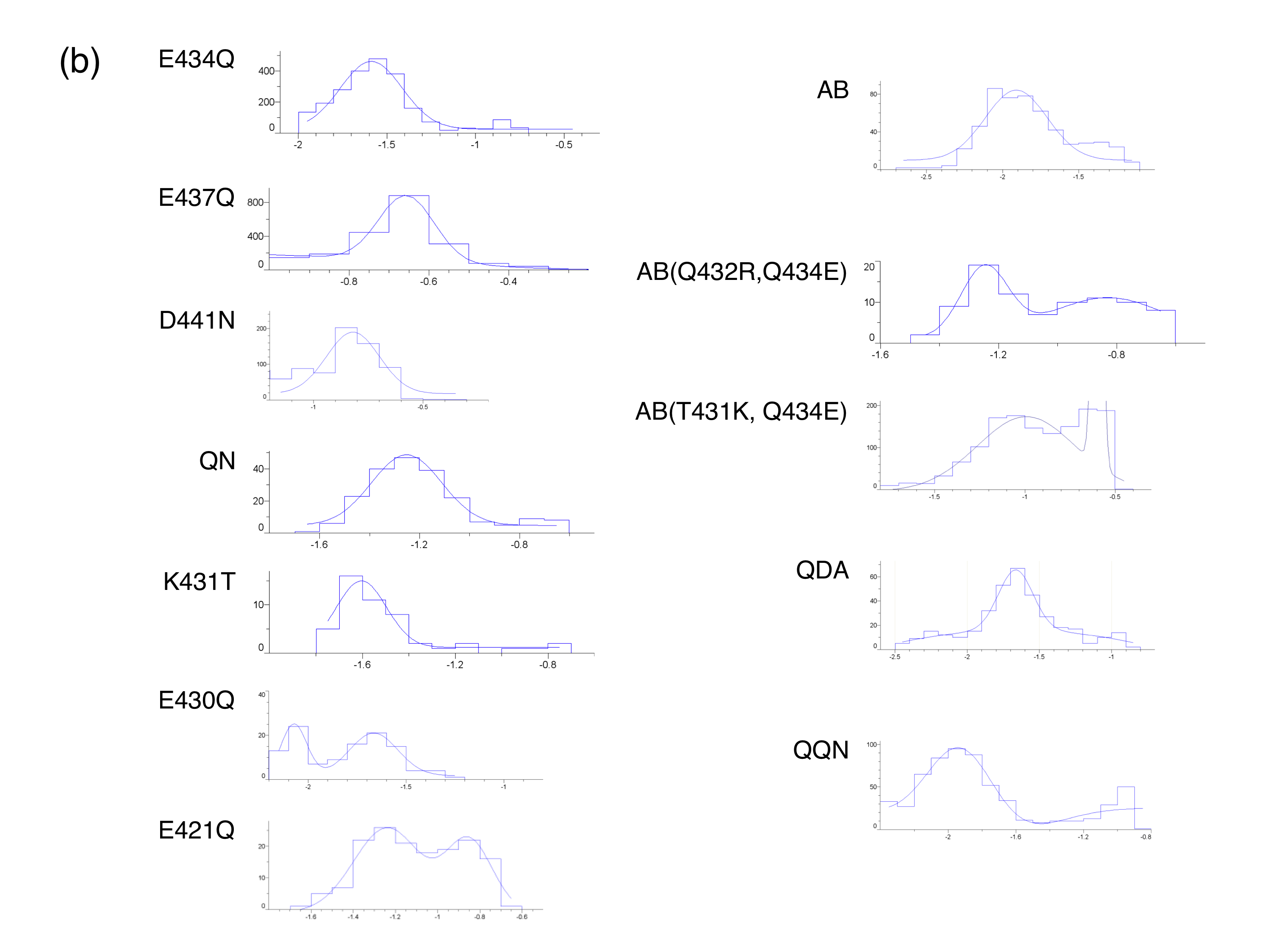

Supplement: Figure S2 — The effect of MA helix mutations on the single channel conductance of 5-HT3A receptors expressed in tsA201 cells. Outside-out patches were excised from cells. The holding potential was –60 mV for all receptor constructs; 5-HT (10 μM) was used to evoke channel activity. Examples of the single channel currents (a) are shown with openings as downward deflections. The amplitude histograms from a single patch (b) are shown with amplitude (pA) plotted on the x-axis and number of events plotted on the y-axis. Conductance was calculated from the average current amplitude from at least three patches and from a minimum of two separate transfections. [file bph0171-1617-sd2.zip › bph_12536_sm_Figure S2b.tiff]

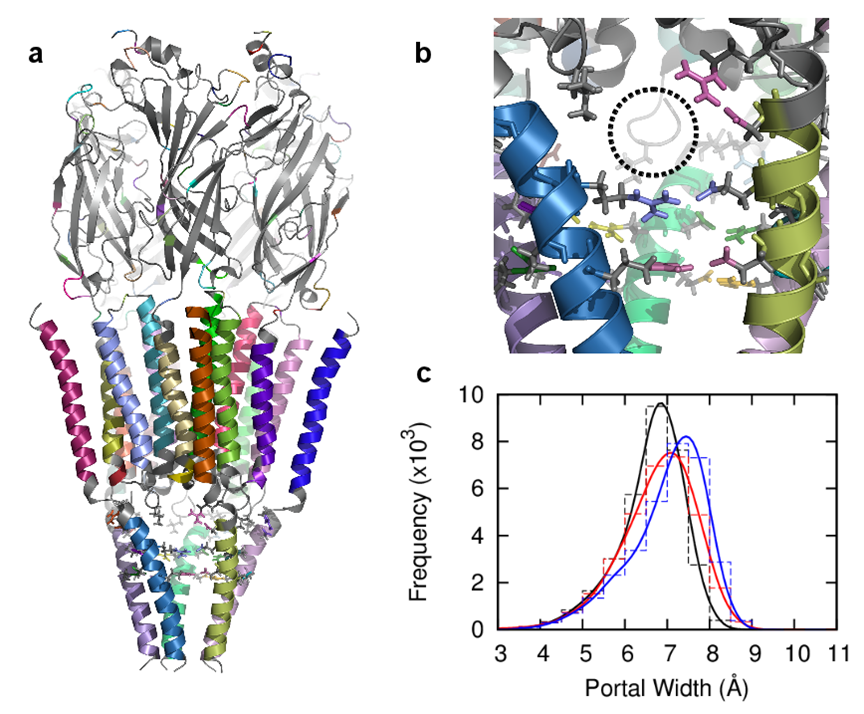

Supplement: Figure S3 — Rigidity analysis of 5-HT3A receptor. (a) Tertiary structure of the 5-HT3A receptor with residues coloured by rigid clusters assigned by the FIRST software. The ECD is relatively flexible, while the TMD and MA helices form large rigid units. (b) Close-up of the portal region. E434, E437 and D441, along with their salt bridge partners, are shown as sticks and form small side chain rigid units, spanning the MA helices in the first two cases. L335 is also shown as the fourth residue lining the portal. The dashed circle denotes the approximate position of the portal. (c) Distribution of portal widths for the three receptors from constrained geometric simulation with Ecut set to-0.6 kcal mol−1. Histograms are fitted with cubic splines. Decreased flexibility leads to narrower portals (compared to Ecut =-1.0 kcal mol−1), while the QQN (red lines) and E434Q (blue lines) mutant receptor portals remain wider than the wild type 5-HT3A receptor portals (black lines). [file bph0171-1617-sd3.tif]

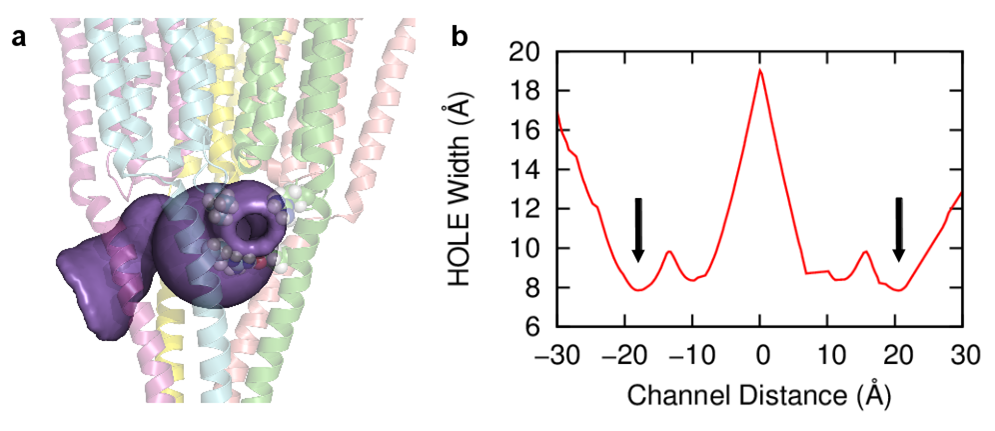

Supplement: Figure S4 — HOLE profiles of the MA portals. (a) Example HOLE channel used to calculate the portal width. Channels through the MA helices are searched for along a line joining the centre of the helices with the centre of a portal (defined by residues L335, R436, E437 and R444, shown as spheres). (b) The channel width is measured at each point along the HOLE profile and the minimum width (denoted by arrows) is assigned to the portal width. The process is repeated for every saved conformation. [file bph0171-1617-sd4.tif]

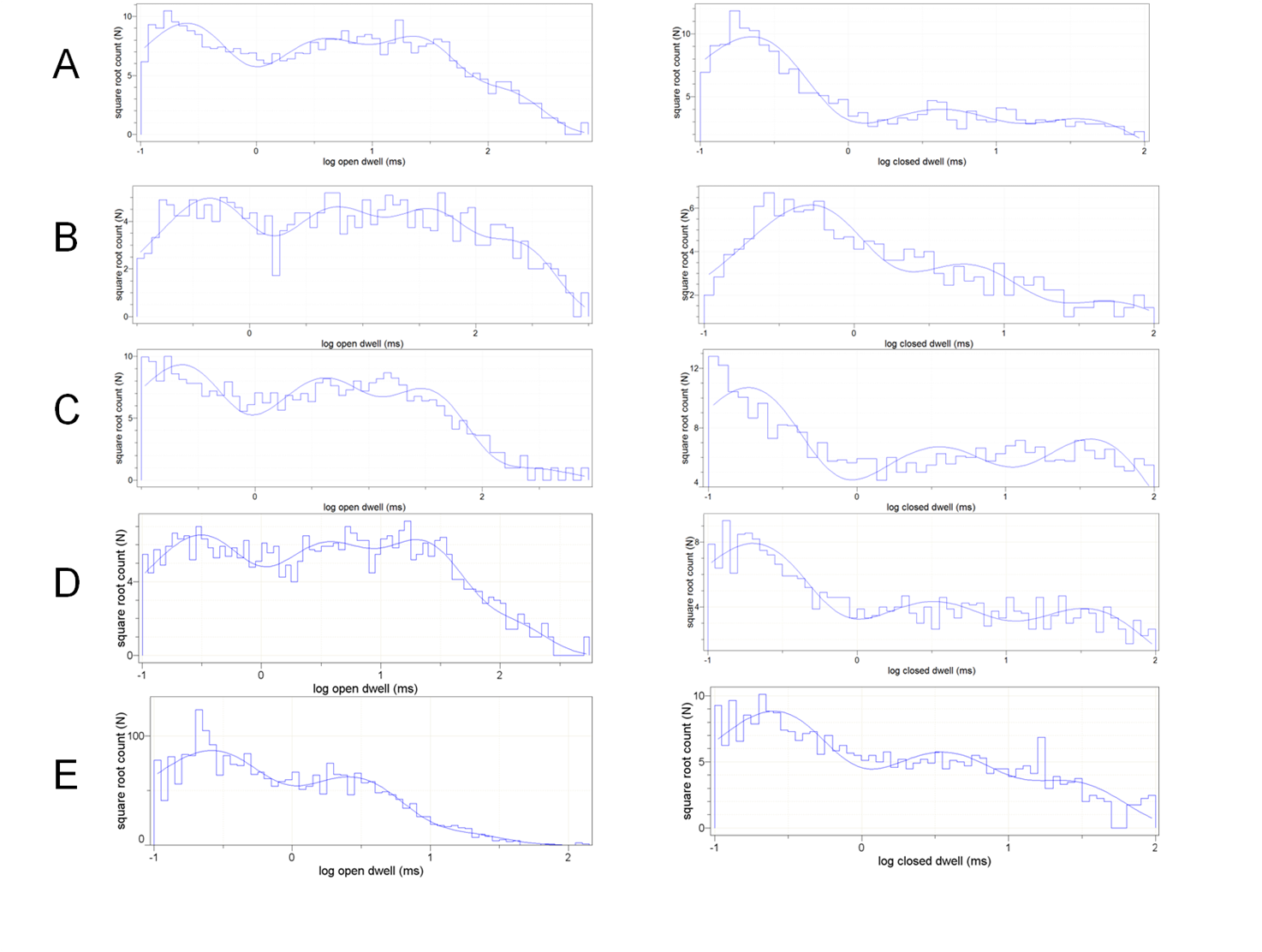

Supplement: Figure S5 — Open and closed dwell duration histograms of the (A) 5-HT3A(QDA) receptor, (B) 5-HT3A(QQN) receptor, (C) 5-HT3AB receptor, (D) 5-HT3AB(Q432R,Q434E) receptor, (E) 5-HT3AB(T431K,Q434E) receptor. 5-HT 10 μM was used to evoke 5-HT3A(QDA) receptor and 5-HT3A(QQN) receptor channel activity. 5-HT 100 μM was used to evoke activity of wild type heteromeric receptors and 5-HT3AB (T431K,Q434E) receptors while 1 mM 5-HT was used for 5-HT3AB(Q432R,Q434E) receptors. Open dwell histograms are on the left and closed dwell histograms are on the right. [file bph0171-1617-sd5.tif]

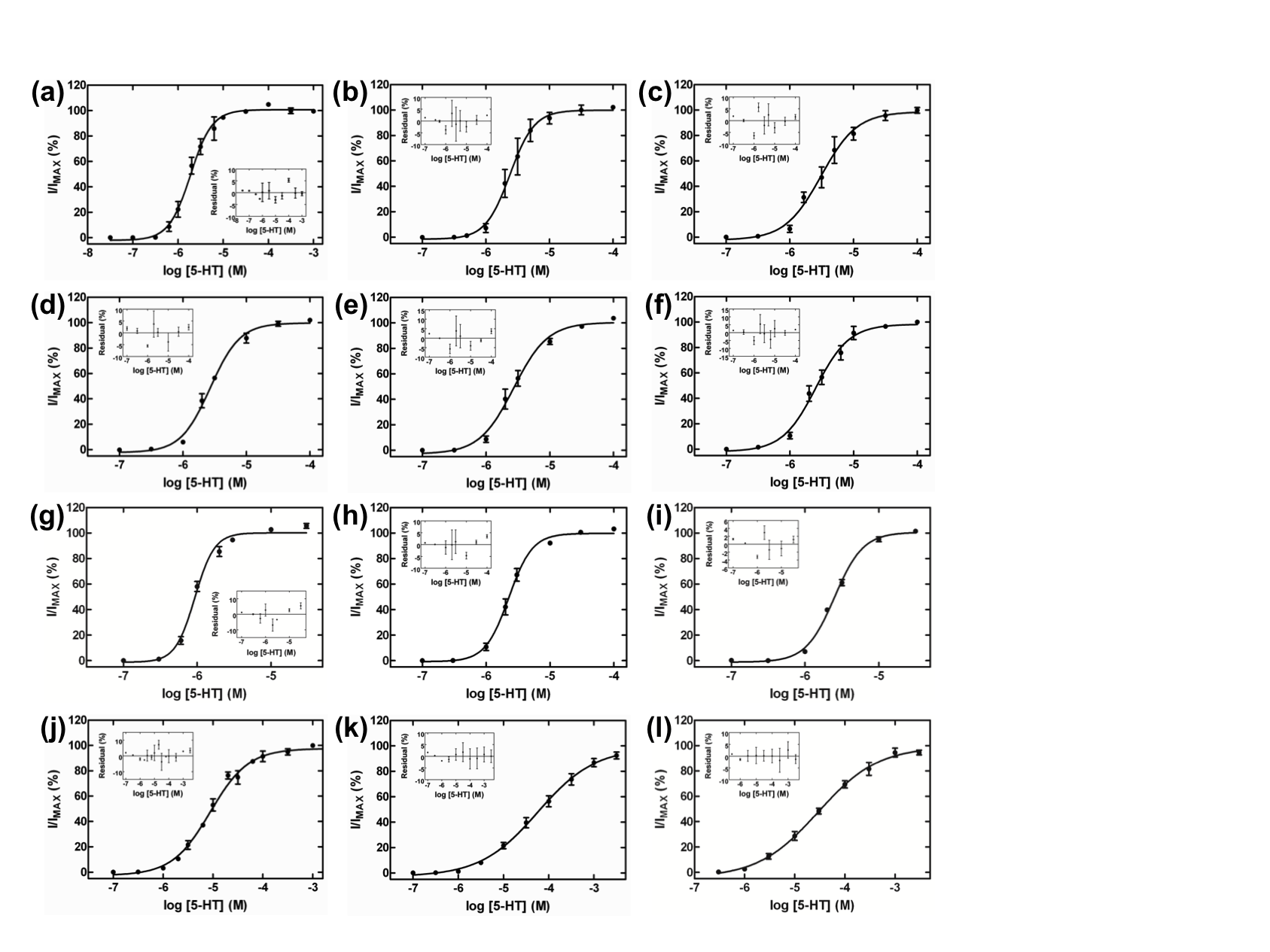

Supplement: Figure S6 — Concentration-response curves of 5-HT activated 5-HT3 receptors expressed in Xenopus oocytes. Inserts show the resulting residual plots obtained from the corresponding concentration-response curve. The data variability confirms there is no pattern consistent with desensitization. Receptor populations: (a) 5-HT3A wild type; (b) 5-HT3A(QDA); (c) 5-HT3A(QQN); (d) 5-HT3A(E437Q, D441N); (e) 5-HT3A(D441N); (f) 5-HT3A (E434Q); (g) 5-HT3A(K431T); (h) 5-HT3A(E430Q); (i) 5-HT3A(E272Q); (j) 5-HT3AB wild type; (k) 5-HT3AB(Q432R,Q434E) and (l) 5-HT3AB(T431K,Q434E). [file bph0171-1617-sd6.tif]

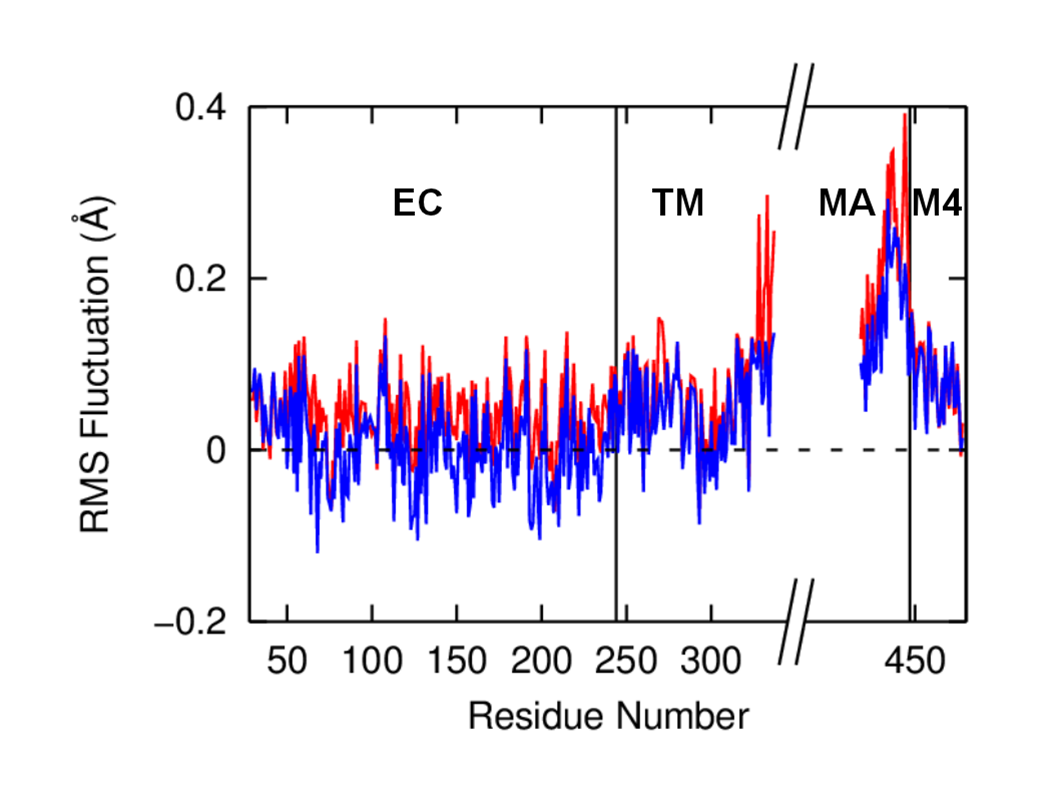

Supplement: Figure S7 — Mutant mobility by residue. Root mean square (RMS) fluctuations of the QQN mutant receptor (red) and E434Q mutant receptor (blue) relative to wild type 5-HT3A receptor by residue. Large increases in flexibility are observed at the top of the MA helices, close to the mutation sites, and at the base of the TM3 helix. [file bph0171-1617-sd7.tif]

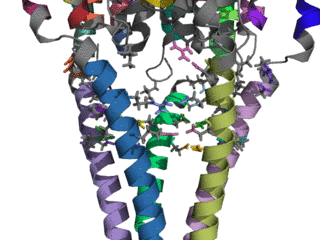

Supplement: Video S1 — Examples of conformations generated by FRODA constrained geometric simulations of the MA portal regions of the wild type 5-HT3A receptor. Colours denote rigid units generated by the FIRST software. Movies generated using Pymol (The PyMOL Molecular Graphics System, Version 1.3 Schrödinger, LLC). [file bph0171-1617-sd8.gif]

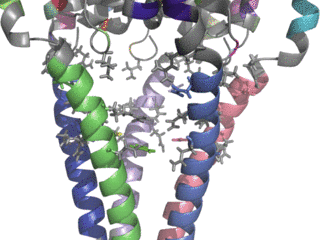

Supplement: Video S2 — Examples of conformations generated by FRODA constrained geometric simulations of the MA portal regions of the 5-HT3A(QQN) receptor. Colours denote rigid units generated by the FIRST software. Movies generated using Pymol (The PyMOL Molecular Graphics System, Version 1.3 Schrödinger, LLC). [file bph0171-1617-sd9.gif]

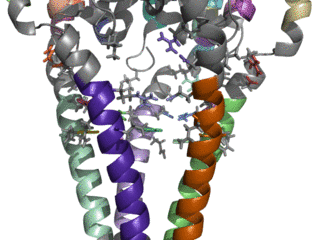

Supplement: Video S3 — Examples of conformations generated by FRODA constrained geometric simulations of the MA portal regions of the 5-HT3A(E434Q) receptor. Colours denote rigid units generated by the FIRST software. Movies generated using Pymol (The PyMOL Molecular Graphics System, Version 1.3 Schrödinger, LLC). [file bph0171-1617-sd10.gif]
